# Regulation of Nitrogen Metabolism by GATA Zinc Finger Transcription Factors in *Yarrowia lipolytica*

**DOI:** 10.1128/mSphere.00038-17

**Published:** 2017-02-15

**Authors:** Kyle R. Pomraning, Erin L. Bredeweg, Scott E. Baker

**Affiliations:** a Energy and Environment Directorate, Pacific Northwest National Laboratory, Richland, Washington, USA; b Environmental Molecular Sciences Laboratory, Pacific Northwest National Laboratory, Richland, Washington, USA; Carnegie Mellon University

**Keywords:** biotechnology, carbon metabolism, lipid synthesis, metabolic regulation, nitrogen metabolism, yeasts, *Yarrowia lipolytica*, nitrogen catabolite repression, carbon catabolite repression, zinc finger, transcription factor, GATA transcription factor, GATA, nitrogen, gene regulation, oleaginous yeast, lipid metabolism

## Abstract

Fungi accumulate lipids in a manner dependent on the quantity and quality of the nitrogen source on which they are growing. In the oleaginous yeast *Yarrowia lipolytica*, growth on a complex source of nitrogen enables rapid growth and limited accumulation of neutral lipids, while growth on a simple nitrogen source promotes lipid accumulation in large lipid droplets. Here we examined the roles of nitrogen catabolite repression and its regulation by GATA zinc finger transcription factors on lipid metabolism in *Y. lipolytica*. Deletion of the GATA transcription factor genes *gzf3* and *gzf2* resulted in nitrogen source-specific growth defects and greater accumulation of lipids when the cells were growing on a simple nitrogen source. Deletion of *gzf1*, which is most similar to activators of genes repressed by nitrogen catabolite repression in filamentous ascomycetes, did not affect growth on the nitrogen sources tested. We examined gene expression of wild-type and GATA transcription factor mutants on simple and complex nitrogen sources and found that expression of enzymes involved in malate metabolism, beta-oxidation, and ammonia utilization are strongly upregulated on a simple nitrogen source. Deletion of *gzf3* results in overexpression of genes with GATAA sites in their promoters, suggesting that it acts as a repressor, while *gzf2* is required for expression of ammonia utilization genes but does not grossly affect the transcription level of genes predicted to be controlled by nitrogen catabolite repression. Both GATA transcription factor mutants exhibit decreased expression of genes controlled by carbon catabolite repression via the repressor *mig1*, including genes for beta-oxidation, highlighting the complex interplay between regulation of carbon, nitrogen, and lipid metabolism.

**IMPORTANCE** Nitrogen source is commonly used to control lipid production in industrial fungi. Here we identified regulators of nitrogen catabolite repression in the oleaginous yeast *Y. lipolytica* to determine how the nitrogen source regulates lipid metabolism. We show that disruption of both activators and repressors of nitrogen catabolite repression leads to increased lipid accumulation via activation of carbon catabolite repression through an as yet uncharacterized method.

## Abstract (precis)

Nitrogen source is commonly used to control lipid production in industrial fungi. Here we identified regulators of nitrogen catabolite repression in the oleaginous yeast *Y. lipolytica* to determine how the nitrogen source regulates lipid metabolism. We show that disruption of both activators and repressors of nitrogen catabolite repression leads to increased lipid accumulation via activation of carbon catabolite repression through an as yet uncharacterized method.

## INTRODUCTION

Fungi are capable of producing a wide variety of lipids valuable as fuel, lubricant, and nutritional and health products and are particularly attractive as an environmentally sustainable replacement for fossil fuel-derived compounds. Study of oleaginous fungi and nonoleaginous fungi has revealed that lipid metabolism is dependent on environmental conditions. Limitation of major nutrients, including nitrogen, oxygen, phosphorus, and sulfur, in the presence of excess carbon has emerged as a reliable means to promote lipid accumulation in a variety of fungi (1–9), and it is well-known that the quantity of nitrogen plays a role in lipid metabolism and has an inverse relationship with lipid accumulation (10–12). The source of carbon and nitrogen also has a strong influence on lipid accumulation. *Yarrowia lipolytica* exhibits dramatically different growth rates and morphologies when grown on different sources of nitrogen, and we have noted that the oleaginous yeasts *Y. lipolytica* and *Lipomyces starkeyi* (David Culley, personal communication) do not accumulate lipids in large lipid droplets during logarithmic-phase growth in complex medium containing peptone as the nitrogen source, suggesting that these yeasts can be grown under conditions that render them nonoleaginous. This phenomenon is not unique to *Y. lipolytica* and is a common feature of metabolism in oleaginous fungi (13). In particular, there is a difference associated with growth on a simple nitrogen source, where biosynthesis of nitrogenous compounds is required, versus growth on a complex nitrogen source, where many nitrogenous compounds are readily available and need only be transported into the cell.

Nitrogen utilization is well studied in yeasts and filamentous fungi and is regulated by the process of nitrogen catabolite repression (NCR) which controls gene expression through a family of GATA binding zinc finger transcription factors (reviewed in references [Bibr B14][Bibr B15][Bibr B21]). Previous work identified DNA sequence motifs containing 5′-GATAA-3′ similar to those bound by GATA zinc finger transcription factors (22, 23) in the promoter regions of genes upregulated when nitrogen is limited in *Y. lipolytica* (24), suggesting that NCR operates in this fungus and may play a role in lipid accumulation. Interestingly, decreased flux through amino acid biosynthetic pathways, regulated by GATA transcription factors and target of rapamycin (TOR), is implicated in regulating carbon flow toward lipid metabolism in *Y. lipolytica* (9). However, NCR has not been studied in *Y. lipolytica*, and the homology of GATA transcription factors in *Y. lipolytica* to those in well-studied model species is unclear. We therefore investigated the effect of nitrogen quality on lipid metabolism and functionally characterized GATA transcription factors in *Y. lipolytica* to examine the link between nitrogen regulation and lipid accumulation and assessed the role of nitrogen source in lipid accumulation.

## RESULTS

### Lipid droplets expand and peroxisomes proliferate when grown on a simple nitrogen source.

**FIG 1:**
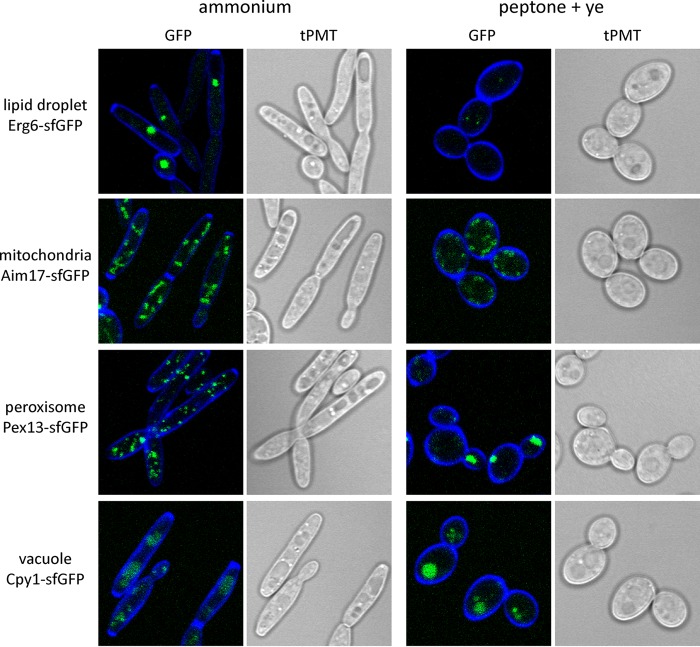
Response of organelle quantity and morphology to nitrogen source. Isogenic *Y. lipolytica* strains bearing sfGFP-tagged organelles were examined during log-phase growth on medium containing either ammonium sulfate or peptone plus yeast extract (peptone + ye) as the nitrogen source. Cells are stained with calcofluor white (blue). tPMT, transmitted light detector.

We cultivated previously characterized, isogenic *Y. lipolytica* strains with superfolder green fluorescent protein (sfGFP)-tagged organelles (25) to observe the effects of simple versus complex nitrogen sources on intracellular structures. Growth with ammonium as the sole nitrogen source results in large lipid droplets, while exponentially growing cells with a complex carbon and nitrogen source accumulate few small lipid droplets (Fig. 1). *Y. lipolytica* growing with peptone and yeast extract maintain a rounded cell morphology, while cells growing with ammonium as the sole nitrogen source are typically elongated. Mitochondria and vacuoles are present in similar quantities regardless of the nitrogen source, while peroxisomes are evident at higher quantities in the elongated cells growing on ammonium as the sole nitrogen source.

### GATA transcription factors control nitrogen utilization in *Yarrowia lipolytica.*

**FIG 2:**
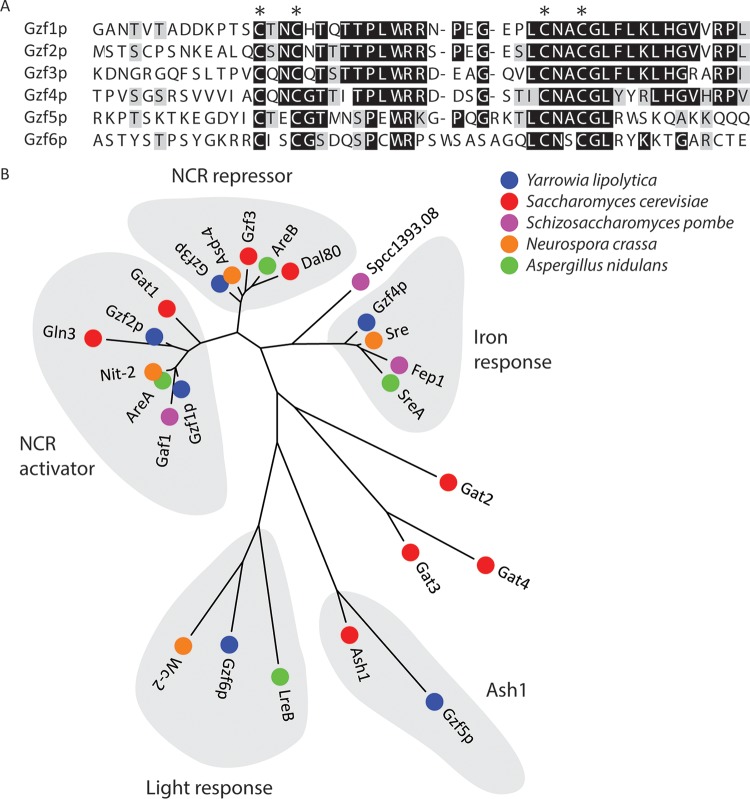
Identification of GATA binding transcription factors in *Y. lipolytica*. (A) Alignment of the DNA interaction domain of GATA-like zinc finger transcription factors from *Y. lipolytica*. Cysteines from the canonical Cys-X_2_-Cys-X_17_-Cys-X_2_-Cys domain are indicated by an asterisk above the sequence alignment. Note that the *Y. lipolytica* proteins clustering with light-responsive transcription factors and Ash1 have X_18_ and X_19_ between the Cys-X_2_-Cys motifs. (B) The DNA interaction domain of GATA transcription factor proteins from fungi with known mutant phenotypes were aligned with predicted *Y. lipolytica* GATA transcription factors using MUSCLE (65) and clustered into families using the neighbor-joining method.

**FIG 3:**
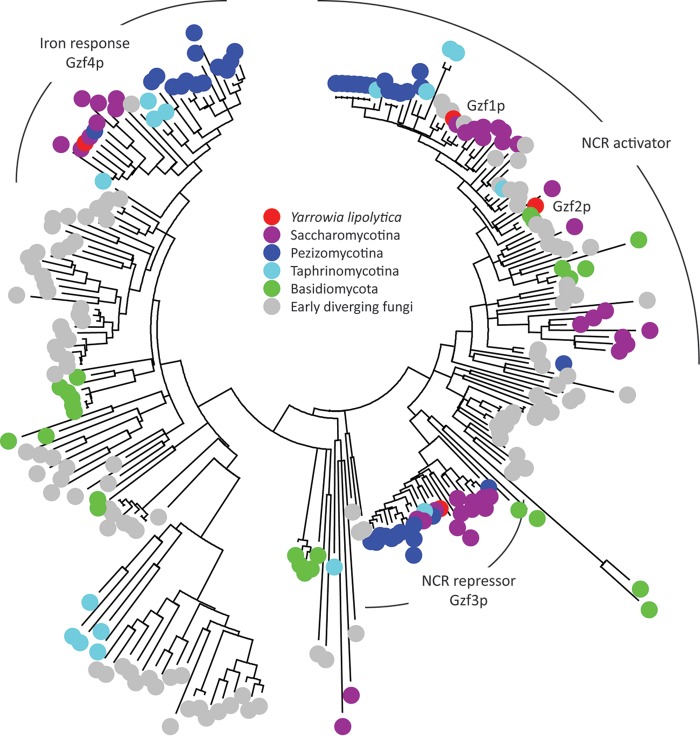
GATA binding transcription factor families in fungi. The DNA interaction domain of GATA zinc finger transcription factors from representative groups of fungi were identified by BLASTp and aligned using MUSCLE. Protein sequences with a Cys-X_2_-Cys-X_4_-Thr-(Pro/Ser)-(Leu/Val)-Trp-Arg-(Arg/Lys)-X_7_-Cys-Asn-X-Cys-X_25_ domain were retained, and duplicate sequences within individual species were removed. Only the best aligning sequence was used in proteins with multiple DNA interaction domains. Clustering was done by the neighbor-joining method. Bars encompass proteins that group by regulatory function. Species were sampled from the *Saccharomycotina* (*Ascoidea rubescens*, *Candida tenuis*, *Dekkera bruxellensis*, *Lipomyces starkeyi*, *Metschnikowia bicuspidate*, *Pichia stipites*, *Saccharomyces cerevisiae*, *Sporopachydermia lactativora*, *Sympodiomyces attinorum*, *Yarrowia lipolytica*, and *Zygoascus hellenicus*), *Pezizomycotina* (*Arthrobotrys oligospora*, *Ascobolus immersus*, *Aspergillus nidulans*, *Botrytis cinerea*, *Fusarium graminearum*, *Mycosphaerella graminicola*, *Neurospora crassa*, *Penicillium chrysogenum*, *Phialocephala scopiformis*, *Stagonospora nodorum*, *Trichophyton rubrum*, *Tuber melanosporum*, and *Xylona heveae*), *Taphrinomycotina* (*Pneumocystis jirovecii*, *Protomyces inouyei*, *Saitoella complicata*, *Schizosaccharomyces japonicus*, *Schizosaccharomyces pombe*, and *Taphrina deformans*), *Basidiomycota* (*Armillaria mellea*, *Cryptococcus neoformans*, *Laccaria bicolor*, *Malassezia sympodialis*, *Meira miltonrushii*, *Melampsora lini*, *Mixia osmundae*, *Paxillus involutus*, *Trametes versicolor*, and *Ustilago maydis*), and various lineages of early diverging fungi (*Allomyces macrogynus*, *Antonospora locustae*, *Basidiobolus meristosporus*, *Batrachochytrium dendrobatidis*, *Catenaria anguillulae*, *Coemansia reversa*, *Conidiobolus coronatus*, *Conidiobolus thromboides*, *Encephalitozoon cuniculi*, *Glomus intraradices*, *Gonapodya prolifera*, *Hesseltinella vesiculosa*, *Linderina pennispora*, *Lobosporangium transversal*, *Mortierella elongata*, *Mortierella multidivaricata*, *Mucor circinelloides*, *Neocallimastix californiae*, *Piromyces finnis*, *Phycomyces blakesleeanus*, and *Spizellomyces punctatus*).

Work in a variety of yeasts and filamentous fungi has found that a family of GATA binding zinc finger transcription factors regulate genes that participate in regulation of nitrogen utilization. Six genes predicted to encode proteins with GATA binding zinc finger domains were identified in the genome of *Y. lipolytica* and named *gzf1* to *gzf6* (*gzf* for GATA zinc finger). Alignment of the DNA interaction domain revealed that four of the GATA-like transcription factors have the canonical Cys-X_2_-Cys-X_17_-Cys-X_2_-Cys domain present in zinc finger transcription factors that bind the GATA consensus sequence (Fig. 2). Three of these proteins are similar to proteins that regulate nitrogen catabolite repression (NCR) in other fungi (reviewed in references 14, 15, and 18), while the fourth is more similar to proteins involved in iron sensing and regulation of siderophore production (26–28). The remaining two proteins are GATA-like in that they are similar to light-responsive transcription factors (LreB and WC-2) and the Rpd3L histone deacetylate complex component Ash1p in *Saccharomyces cerevisiae*. The GATA factor clustering with light-responsive GATA transcription factors has X_18_ rather than X_17_ between the Cys-X_2_-Cys motifs in *Y. lipolytica* as in the DNA interaction domain of WC-2 from *Neurospora crassa* (29). In *N. crassa*, WC-2 interacts with a GATA-like consensus sequence YCGAT (30), suggesting that the difference in the DNA binding motif changes the specificity of the transcription factor and that the difference is likely to be conserved in *Y. lipolytica*. The GATA-like domain from *S. cerevisiae* Ash1p has X_20_ between the Cys-X_2_-Cys motifs (31), while *Y. lipolytica* has X_19_. The additional residues between the Ash1p Cys-X_2_-Cys motif bind the GATA-like consensus sequence YTGAT in *S. cerevisiae* (32). Thus, the *Y. lipolytica* genome encodes four genuine GATA binding zinc finger proteins (*gzf1*, Yali0D20482g; *gzf2*, Yali0F17886g; *gzf3*, Yali0C22682g; *gzf4*, Yali0E05555g) and two GATA-like binding proteins (*gzf5*, Yali0E16577g; *gzf6*, Yali0E05346g) (Fig. 2). True GATA transcription factors (defined here as having a Cys-X_2_-Cys-X_17_-Cys-X_2_-Cys motif) are found in all major fungal lineages (Fig. 3), but they have been studied genetically in only a small number of model organisms. It is interesting to note that while those that control NCR and siderophore biosynthesis are well conserved across evolutionary space, additional groups of proteins with GATA zinc finger domains are conserved in specific lineages and have yet to be characterized functionally.

**FIG 4:**
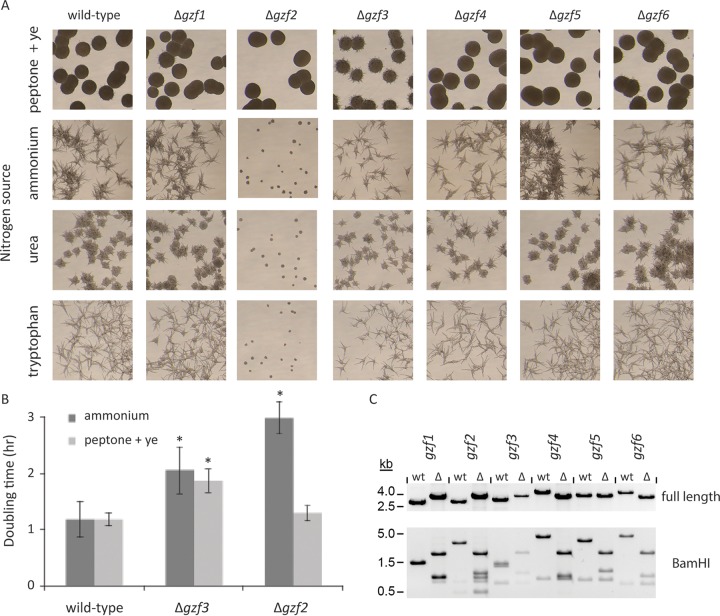
Phenotypes of *Y. lipolytica* GATA transcription factor mutants on various nitrogen sources. The genes encoding GATA transcription factors in *Y. lipolytica* were replaced with *ura3*. (A) Colony morphology on agar plates after 24-h growth of mutants on YNB-glucose medium (yeast nitrogen base with glucose) with either a simple (ammonium sulfate, urea, or tryptophan) or complex (peptone plus yeast extract [ye]) nitrogen source. Note that the *gzf2* mutant grows poorly on simple nitrogen sources, while the *gzf3* mutant grows more slowly and produces more hyphae on peptone, which suggest that these transcription factors regulate nitrogen utilization. (B) The log-phase growth rate for mutants with nitrogen utilization phenotypes was determined in YNB-glucose medium with either ammonium or peptone and yeast extract as the nitrogen source. The *gzf3* mutant grows more slowly on both nitrogen sources, while the *gzf2* mutant grows more slowly only when ammonium is the sole nitrogen source. Values that are significantly different (*P* < 0.05) from the value for the wild-type strain are indicated by an asterisk. (C) Deletion of GATA transcription factors was confirmed by PCR with primers flanking the genes followed by BamHI digestion. The genes in the wild type (wt) and deletion mutant (Δ) are shown.

We deleted each of the six GATA transcription factor genes in *Y. lipolytica* by replacement with a *ura3*^+^ nutritional marker to determine which genes are responsible for regulating nitrogen metabolism genes in *Y. lipolytica* (Fig. 4C). The deletion mutants were tested for utilization of a variety of single sources of nitrogen, including ammonium, tryptophan, and urea, as well as a complex nitrogen source consisting of peptone and yeast extract (Fig. 4A). Deletion of *gzf2* resulted in a severe growth defect when grown with ammonium, tryptophan, or urea as the sole nitrogen source (Fig. 4A), suggesting that nitrogen utilization is defective in this strain regardless of the source. When grown on a complex nitrogen source (peptone and yeast extract) that provides a wide variety of nitrogenous compounds, this strain grows similarly to the wild-type strain both on solid and liquid cultures (Fig. 4), suggesting that *gzf2* is required for expression of genes normally repressed by NCR. Its phenotype is consistent with positive regulation of NCR like that of *gln3* and *gat1* which in *S. cerevisiae* are sequestered in the cytosol when the cells are grown on a rich nitrogen source. When nitrogen becomes limiting for *S. cerevisiae*, *gln3* and *gat1* move to the nucleus and activate genes containing upstream GATAA sites (33–36). Deletion of *gzf3* resulted in slight growth defects both on single and complex nitrogen sources and increased hyphal formation on a complex nitrogen source (Fig. 4A), consistent with its predicted function as a negative regulator of NCR. Interestingly, deletion of *gzf1*, which is most similar to AreA of *Aspergillus nidulans* and Nit-2 of *N. crassa*, which are activated in response to nitrogen starvation (37, 38), did not display a growth defect on any of the nitrogen sources tested. This is not unprecedented and was also found for mutation of the *Schizosaccharomyces pombe* ortholog, *gaf1* (39).

Gzf1p and Gzf2p have similar DNA binding domains (Fig. 2), but sequence alignment clusters Gzf1p with activators of genes repressed by NCR in filamentous *Pezizomycotina* fungi and other *Saccharomycotina* yeasts with the notable exception of *S. cerevisiae* (Fig. 3), while Gzf2p shows more differences. Many of the *Saccharomycotina* species investigated have two proteins that group with activators of NCR: one that is similar to those found in the *Pezizomycotina* and one that is similar to Gln3p from *S. cerevisiae* (Fig. 3). Our phenotypic analysis suggests that the more diverged transcription factor (*gzf2*) is essential for growth on simple nitrogen sources, while the function of *gzf1* remains to be defined.

Another striking phenotype in the GATA collection was the susceptibility of the Δ*gzf3* and Δ*gzf2* nitrogen regulator mutants and the homolog of the siderophore biosynthesis regulator Δ*gzf4* to 4 mM hydrogen peroxide (see Fig. S1 in the supplemental material). Interestingly, deletion of *gzf6*, homolog of the *N. crassa wc-2* gene, exhibited resistance to 6 mM hydrogen peroxide. The *N. crassa* ortholog WC-2 is cyclically moderated by reactive oxygen species, and it regulates several players in reactive oxygen species homeostasis (40), suggesting conservation of regulatory function between these species.

10.1128/mSphere.00038-17.1 FIG S1  Sensitivity of *Y. lipolytica* GATA transcription factor mutants to reactive oxygen species. *Y. lipolytica* strains were pregrown overnight in yeast extract-peptone-dextrose (YPD) and then diluted to an OD_600_ of 0.4, followed by 10-fold serial dilutions. Plates with 4 or 6 mM hydrogen peroxide were spotted with 3.5 µl of culture and incubated for 120 h at 28°C prior to imaging. Images were captured on a dissection microscope at a magnification of 4× (YNB) or 8× (4 and 6 mM H_2_O_2_). Note the small Δ*gzf6* colonies growing at 6 mM H_2_O_2_. Download FIG S1, PDF file, 0.1 MB. Copyright © 2017 Pomraning et al. 2017 Pomraning et al. This content is distributed under the terms of the Creative Commons Attribution 4.0 International license.

### Disruption of nitrogen utilization regulators leads to lipid accumulation.

**FIG 5:**
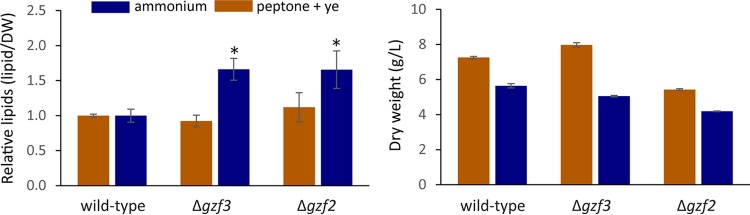
Lipid content of GATA transcription factor mutants. *Y. lipolytica* strains were grown in batch culture conditions for 5 days, after which lipids were extracted and quantified. Lipid quantities as a fraction of dry weight (DW) are normalized to the wild-type strain independently for each nitrogen source. Asterisks indicate significant differences in relative lipid content between the wild-type strain and a mutant strain for a given nitrogen source (*P* < 0.05). Error bars indicate the standard deviations from triplicate measurements.

We are particularly interested in whether disruption of nitrogen regulators would impact lipid metabolism by either artificially limiting the cells for nitrogenous metabolites due to failure to properly regulate nitrogen metabolism or alternatively by the failure to sense and signal the intracellular nitrogen state. To that end, we performed a batch lipid accumulation experiment by cultivating each mutant and the wild-type strain for 5 days at which point the lipids were extracted and quantified. When growing on peptone plus yeast extract as the nitrogen source, neither the *gzf3* nor *gzf2* mutant accumulated significantly different amounts of lipids. However, when grown on ammonium sulfate as the sole nitrogen source, both mutants accumulated nearly twice the concentration of lipids on a gram of lipid per gram (dry weight) basis (Fig. 5). Greater accumulation of lipids in the mutants when growing on ammonium is reflected in part by their lower total biomass accumulated. Growth on peptone plus yeast extract as the nitrogen source yields higher biomass after batch cultivation in all strains analyzed; however, the Δ*gzf3* mutant yielded greater biomass than the wild-type strain did (Fig. 5). This may reflect less production of secreted extracellular metabolites, such as citrate, which would have been removed with the culture flowthrough for determination of the weight (dry weight) in this strain. Superior accumulation of lipids on a simple nitrogen source, such as ammonium sulfate, is promising for biotechnological development of nitrogen assimilation mutants with improved lipid production.

### Transcriptional response to simple versus complex nitrogen sources.

**FIG 6:**
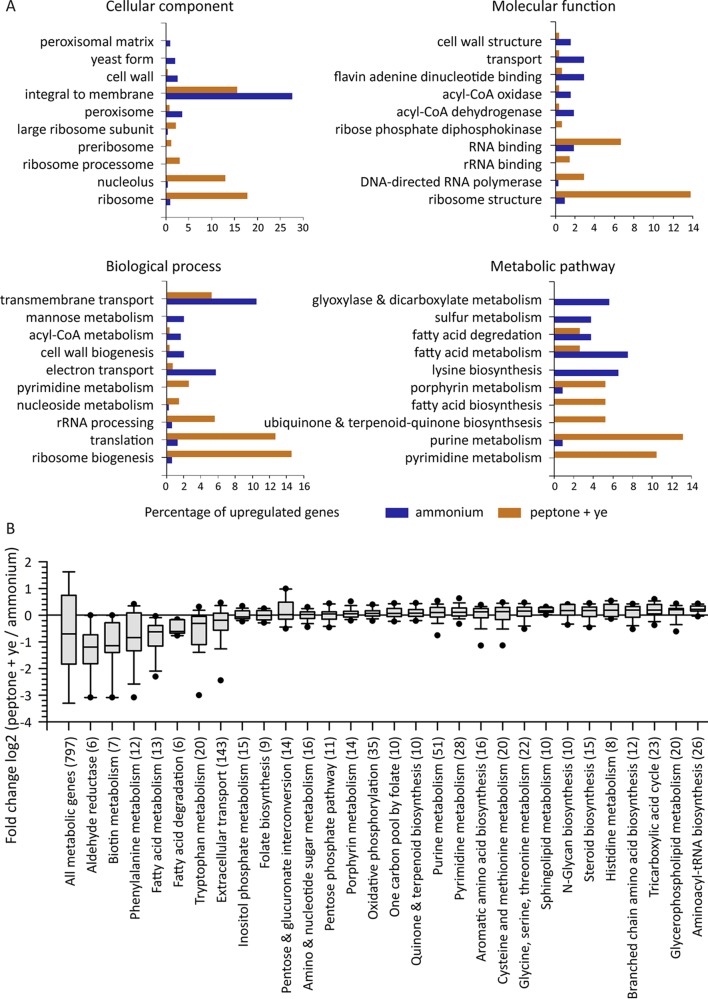
Gene Ontology analysis of changes in expression of *Y. lipolytica* grown with ammonium sulfate or peptone plus yeast extract as the nitrogen source. (A) The 500 most upregulated genes in *Y. lipolytica* grown on each nitrogen source were compared to a background model of all quantified genes to identify enriched Gene Ontology (GO) categories. An additional metabolic pathway category was constructed and analyzed using terms from *Y. lipolytica* metabolic model 1508190002 (9). (B) Distribution of fold change values for metabolic pathway categories. Note that metabolic genes in general are more highly expressed in cells grown with ammonium as the nitrogen source. The number of genes in each category is shown in parentheses.

**FIG 7:**
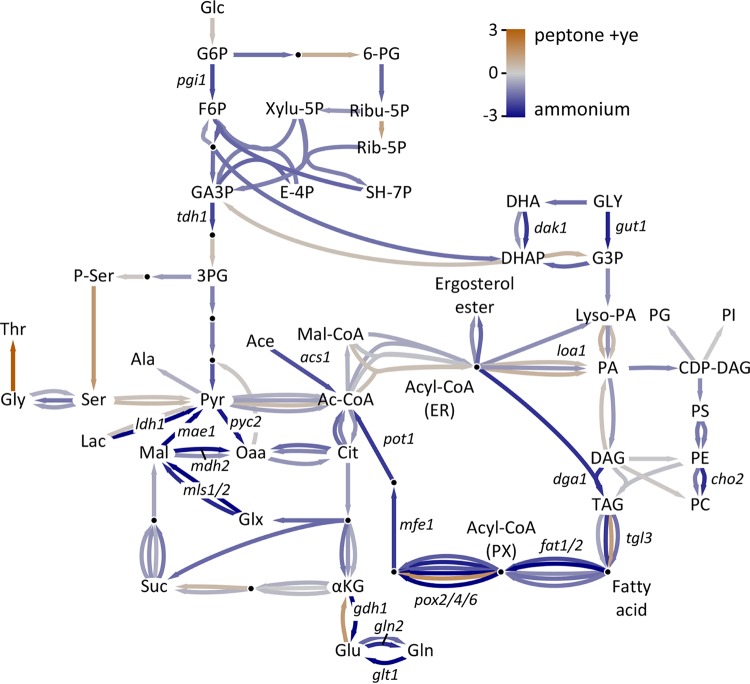
Response of metabolism to nitrogen source. Log_2_ (peptone plus yeast extract/ammonium) expression fold change values from RNA sequencing overlaid on a curated metabolic map for *Y. lipolytica*. Genes with an expression value in cells grown on ammonium greater than eightfold that of cells grown on peptone plus yeast extract are indicated. Abbreviations: G6P, glucose-6-phosphate; F6P, fructose-6-phosphate; P-Ser, phosphorylated serine; Oaa, oxaloacetate; αKG, α-ketoglutarate; Ac-CoA, acetyl coenzyme A; ER, endoplasmic reticulum; DAG, diacylglycerol; PI, phosphatidylinositol; GA3P, glyceraldehyde 3-phosphate; 6-PG, 6-phosphogluconate; Ribu-5P, ribulose-5-phosphate; Rib-5P, ribose-5-phosphate; SH-7P, sedoheptulose 7-phosphate; E-4P, erythrose 4-phosphate; 3PG, 3-phosphoglycerate; Xylu-5P, xylulose-5-phosphate; DHA, dihydroxyacetone; DHAP, dihydroxyacetone phosphate; Lyso-PA, lysophosphatidic acid; PG, phosphatidylglycerol; PS, phosphatidylserine; PE, phosphatidylethanolamine; PC, phosphatidylcholine; DAG, diacylglycerol; TAG, triacylglycerol; PI, phosphatidylinositol.

It is clear that drastic changes take place in response to the source of carbon and nitrogen, particularly at the lipid droplets and peroxisomes. To better understand the changes occurring, we sequenced RNA from log-phase cells (wild-type, Δ*gzf3*, and Δ*gzf2* cells) growing on either the simple nitrogen source (ammonium sulfate) or the complex nitrogen source (peptone plus yeast extract). Differentially expressed genes were determined for each strain growing on the different nitrogen sources as well as between the wild-type and mutant strains on a given nitrogen source to determine the nitrogen assimilation relevant regulon of the GATA transcription factor mutants (Data Set S1). Gene Ontology (GO) analysis revealed that genes associated with the ribosome and translation as well as purine and pyrimidine metabolism are overrepresented in upregulated genes when the cells are grown on peptone plus yeast extract as the nitrogen source, while upregulated genes in cells grown on ammonium are associated with carbon and nitrogen metabolism (Fig. 6A). We analyzed lipid metabolism (41, 42) in particular to determine how nitrogen quality directs carbon toward storage lipids when the cells are grown with ammonium as the sole nitrogen source. Unexpectedly, we found that the genes encoding lipid droplet-targeted lipases involved in mobilization of triglycerides (*tgl1*, Yali0E32035g; *tgl2*, Yali0E31515g; *tgl4*, Yali0F10010g) as well as predicted acyl coenzyme A (acyl-CoA) synthases (*faa1*, Yali0D17864g; *faa2*, Yali0C05885g; *faa3*, Yali0E20405g; *fat1*, Yali0E16016g; *fat2*, Yali0C09284g) and genes involved in beta-oxidation (*pox1*, Yali0E32835g; *pox2*, Yali0F10857g; *pox3*, Yali0D24750g; *pox4*, Yali0E27654g; *pox6*, Yali0E06567g; *mfe1*, Yali0E15378g; *pot1*, Yali0E18568g) are upregulated in cells grown on ammonium relative to cells grown on peptone plus yeast extract (Fig. 7). The presence of greater numbers of peroxisomes in cells grown on ammonium (Fig. 1) demonstrates that large lipid droplets are formed even as storage lipids are being utilized to provide cellular energy. This suggests that large lipid droplets are not formed in peptone plus yeast extract because the rate of storage lipid synthesis is reduced rather than being rapidly turned over via beta-oxidation.

10.1128/mSphere.00038-17.2 DATA SET S1  Expression quantification by RNA-seq. Data from six experimental conditions were aligned to the *Y. lipolytica* transcript models and quantified. This summary includes FPKM values from the six conditions as well as log_2_ fold change values and statistical values for a selection of strain and condition comparisons. Download DATA SET S1, XLSX file, 1.1 MB. Copyright © 2017 Pomraning et al. 2017 Pomraning et al. This content is distributed under the terms of the Creative Commons Attribution 4.0 International license.

**FIG 8:**
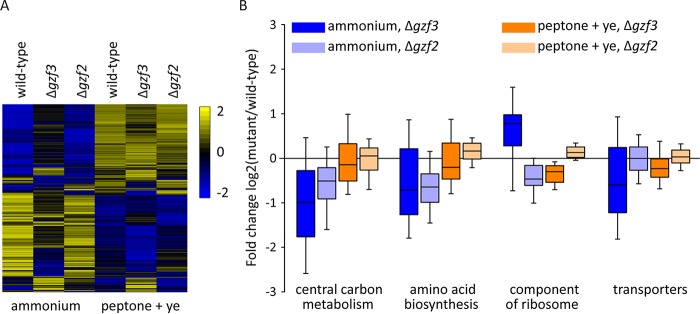
Deletion of GATA transcription factors categorically alters gene expression. (A) Row normalized average log_2_ fragments per kilobase of exon model per million mapped fragments (FPKM) values for each gene are clustered according to the centroid linkage method using the Spearman distance metric. (B) Expression level differences between each of the two mutants and the wild-type strain were determined for all genes in cells grown with either ammonium or peptone and yeast extract as the nitrogen source. The distribution of fold change values between the mutants and the wild type was determined for genes binned into categories based on their function. A positive fold change indicates a higher expression level in the mutant strain, while a negative fold change indicates a higher expression level in the wild type.

We compared the distribution of fold change values from genes belonging to 55 metabolic pathways from a previously published *Y. lipolytica* metabolic model (9) to fold change values from all genes to determine which aspects of metabolism are specifically affected. Interestingly, this analysis found that the total set of metabolic genes analyzed (797 genes) is significantly upregulated when ammonium sulfate is the sole nitrogen source (*P* < 1E−11), as would be expected for cells that must construct the vast majority of their metabolites from glucose and ammonium instead of being able to transport and recycle metabolites present in peptone and yeast extract (Fig. 6B). In general, the transcription level is more drastically altered in the Δ*gzf3* mutant, while overall, the transcription level in the Δ*gzf2* mutant is more similar to that of the wild type (Fig. 8A). Both GATA transcription factor mutants are specifically impaired in their expression of genes important for central carbon metabolism, amino acid biosynthesis, and fatty acid metabolism when grown with glucose and ammonium as the sole carbon and nitrogen sources (Fig. 8B).

The most highly upregulated gene in cells grown on ammonium that directly produces triglycerides is the diacylglycerol acyltransferase *dga1* gene, which is expressed 14-fold higher in cells grown on ammonium than in cells grown on peptone plus yeast extract. This result suggests that the substrate for triglyceride production when ammonium and glucose are the sole nitrogen and carbon sources is diacylglycerol (43–45). Diacylglycerol is formed from phosphatidic acid via dephosphorylation by *pah1* which is upregulated in cells grown on ammonium, while the reverse reaction, catalyzed by *dgk* (42), is expressed more highly in cells grown on peptone plus yeast extract (Fig. 7); thus, the balance of flux toward triglycerides from phosphatidic acid is likely higher in cells grown on ammonium. In *S. cerevisiae*, Pah1p is highly regulated by phosphorylation (46, 47) and required for lipid droplet formation (48) and represents a major regulation point in directing phospholipid versus storage lipid biosynthesis, a feature that appears conserved in *Y. lipolytica*. A number of enzymes have been found to catalyze acylation of lysophosphatidic acid from a variety of substrates in *S. cerevisiae* and include *slc1*, *loa1*, and *ale1* (49–52). Of these three, only the homolog of *loa1* is upregulated in *Y. lipolytica* grown on ammonium (Fig. 7), consistent with a role in production of storage lipids, as has been confirmed by analysis in *S. cerevisiae* (51). Loa1p has not been biochemically characterized in *Y. lipolytica* but does localize to the endoplasmic reticulum (25), consistent with a role in lipid metabolism. Overexpression of *slc1* has been successfully used to increase lipid accumulation in *Y. lipolytica*, and our results suggest that *loa1* may also be a good candidate for this function.

### GATA transcription factor expression is responsive to nitrogen source and regulated by GATA factors.

**FIG 9:**
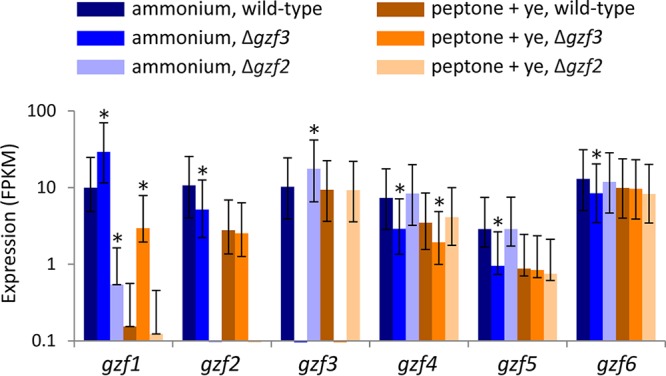
Expression of GATA transcription factors. Relative mRNA expression level of the GATA transcription factors in fragments per kilobase of exon model per million mapped fragments (FPKM). Asterisks indicate a significant difference in expression between the wild-type strain and either of the mutant strains for a given GATA transcription factor (false-discovery rate [*q*] of <0.001). Error bars indicate the upper and lower bounds of the 95% confidence interval.

We are interested in how transcription factors that are expected to interact with similar DNA sequences can have such drastically different phenotypes. Previous work on GATA binding proteins has demonstrated that activators and repressors of NCR can compete for similar binding sites and are regulated by the levels of expression and modifications as well as interacting partners that sequester the GATA binding transcription factor such that they cannot compete for the binding sites (22, 53). The levels of expression of *gzf1*, *gzf2*, *gzf4*, and *gzf5* are increased when the cells are grown on ammonium as the sole nitrogen source, while the level of expression of *gzf3* is not significantly affected by the nitrogen source (Fig. 9). The effect on expression of *gzf1*, for which we did not detect a deletion phenotype, is most dramatic and is affected negatively by deletion of *gzf2* and positively by deletion of *gzf3*. This transcription factor is most similar to the activator present in filamentous fungi (AreA and Nit-2), while the activator that appears to have a significant, phenotypically observable function is more similar to those from *S. cerevisiae* (Gln3p and Gat1p).

### Genes with GATAA DNA motifs in their promoter are repressed by *gzf3.*

**FIG 10:**
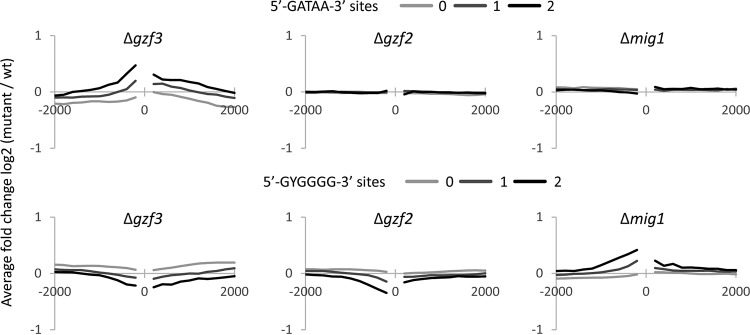
Effect of transcription factor deletion on expression of genes with specific DNA motifs near their transcription start site. The number of GATAA and GYGGGG motifs on each strand of DNA was determined (from 0 to 2 sites) between the transcription start site (labeled 0) and a given distance. The given distances shown are 200 to 2,000 bp in 200-bp intervals, both up- and downstream of the transcription start site. For each interval, the average difference in expression between each transcription factor mutant and wild-type strain is shown. Note that the presence of more GATAA motifs close to the transcription start site is associated with increased expression in the Δ*gzf3* strain, while the presence of GATAA sites near the transcription start site has no effect on expression in the Δ*gzf2* strain.

The canonical binding site for GATA-like transcription factors in yeast is the DNA motif 5′-GATAA-3′ (22, 23). We investigated the association of this motif with changes in transcription of genes in the Δ*gzf3* and Δ*gzf2* strains. Intriguingly, genes with more GATAA sites upstream of the transcription start site tend to be upregulated in the Δ*gzf3* mutant, while the presence of GATAA sites has no effect on transcription level in the Δ*gzf2* mutant (Fig. 10). This suggests that Gzf3p is a general repressor of genes with GATAA sites in their promoter region and that Gzf2p does not generally control the transcription of genes with GATAA sites.

We characterized the types of genes that are upregulated and have GATAA sites in their promoters, as these are likely to represent the major significant biological function(s) that are repressed by Gzf3p. We compared Gene Ontology terms for upregulated genes with and without GATAA motifs in their promoter regions. Upregulated genes without a GATAA motif near their transcription start site are particularly enriched for ribosomal genes, while those with GATAA motifs are enriched for genes involved in amino acid and nucleotide metabolism. This suggests that upregulation of ribosomal genes (Fig. 8B) is a secondary effect and not directly regulated by Gzf3p.

### *gzf2* is required for expression of nitrogen assimilation genes.

*Y. lipolytica* must utilize nitrogen through a handful of well-defined metabolic pathways when growing on ammonium as the sole nitrogen source. The nitrogen assimilation genes *gdh1* (Yali0F17820g), *gdh2* (Yali0E09603g), *gln1* (Yali0F00506g), *gln2* (Yali0D13024g), and *glt1* (Yali0B19998g) all respond significantly to the source of nitrogen. *gdh1*, *gln1*, *gln2*, and *glt1* are upregulated when ammonium is the sole source of nitrogen, while *gdh2* is expressed to a higher level when peptone and yeast extract are the nitrogen source (Fig. 7), suggesting that *Y. lipolytica* degrades glutamate as a major source of nitrogen from peptone plus yeast extract to produce ammonia for glutamine (54). *gdh1* is the most highly upregulated nitrogen assimilation gene when the cells are grown on ammonium, pointing to glutamate synthesis from alpha-ketoglutarate as the main path for nitrogen assimilation under this condition.

**FIG 11:**
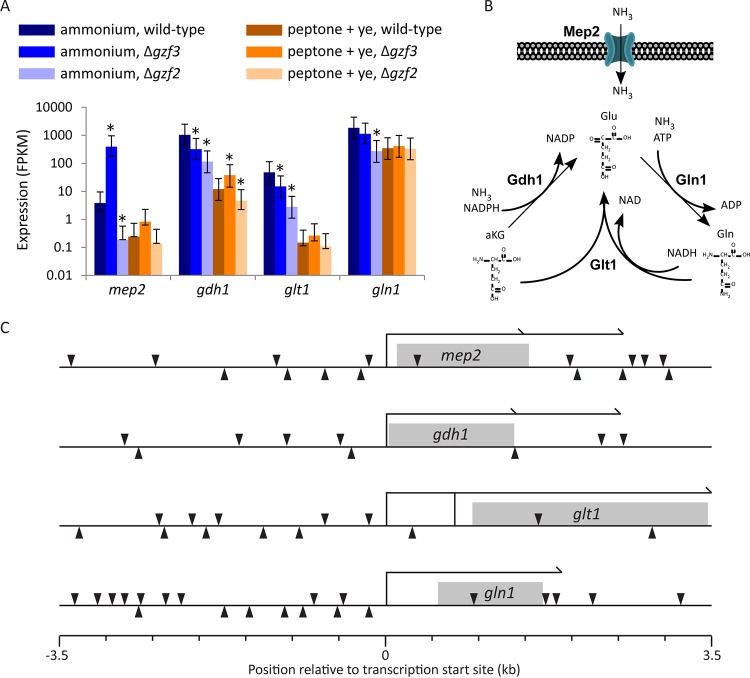
GATA transcription factors control ammonium assimilation genes. (A) Relative mRNA expression level of nitrogen assimilation genes. Asterisks indicate a significant difference in expression between the wild-type strain and either one of the mutant strains (*q* < 0.001). Error bars indicate the upper and lower bounds of the 95% confidence interval. (B) Predicted pathways for transport and assimilation of ammonia in *Y. lipolytica*. aKG, α-ketoglutarate. (C) GATAA sites on both DNA strands relative to the transcription start site of genes are indicated by black arrowheads. Predicted transcription start sites (from alignment of transcriptome sequencing [RNA-seq] data) are designated by bars, while predicted polyadenylation sites are shown as thin long half arrows. RNA-seq data indicate multiple polyadenylation sites for *mep2* and *gdh1*, while *glt1* may have multiple transcription start sites. Coding sequences are indicated by gray boxes.

Inspection of genes that are not activated when the Δ*gzf2* mutant is growing on ammonium as the sole nitrogen source points to a severe reduction in expression of genes essential for nitrogen assimilation from ammonium, namely, *mep2* (Yali0E27203g), *gdh1*, *glt1*, and *gln1* (Fig. 11). These genes are all expressed at a significantly higher level in wild-type *Y. lipolytica* growing on ammonium compared to *Y. lipolytica* growing on peptone plus yeast extract, and therefore, these genes represent the major route for ammonium transport and assimilation, inactivation of which should phenotypically copy nitrogen starvation, and likely represents the cause of the growth defect that this strain exhibits on ammonium but not peptone plus yeast extract. All four of these genes are enriched for GATAA sites near (and not so near) their transcription start site, indicative of regulation by GATA binding transcription factors (Fig. 11C). While deletion of *gzf3* is associated with upregulation of genes with GATAA sites in their promoters, only *mep2* is significantly upregulated in cells grown on ammonium, while in cells grown on peptone plus yeast extract, all four genes are upregulated in the Δ*gzf3* mutant, though to a less significant degree, indicative of their derepression under this condition. On the other hand, *gzf2* appears to be required for activation of these genes in cells grown on ammonium, as all four genes are expressed at a significantly lower level in the Δ*gzf2* mutant grown on ammonium, while in cells grown on peptone plus yeast extract, only *gdh1* is affected. A caveat of this interpretation is that the Δ*gzf2* mutant exhibits a severe growth defect when growing on urea or tryptophan (Fig. 4A) as well as adenine, uracil, lysine, and serine (data not shown) as the sole nitrogen source. Growth defects on a variety of nitrogen sources demonstrate that lack of ammonium transport is not solely responsible and that poor growth may be associated more generally with the transamination reactions required to mobilize intracellular nitrogen.

### Disruption of nitrogen utilization increases carbon catabolite repression.

Gzf2p may specifically activate a small subset of genes via interaction with GATAA sites or via different or less specific DNA motifs. To test these hypotheses, we investigated the promoter regions of genes that are the most up- or downregulated in the GATA transcription factor mutants and, intriguingly, identified enrichment for carbohydrate response elements 5′-GYGGGG-3′ (55, 56) upstream of genes that are downregulated in Δ*gzf2* and Δ*gzf3* mutants grown on both nitrogen sources (Fig. 10). Specifically enriched motifs were not identified in genes upregulated in the Δ*gzf2* mutant. GO analysis revealed that genes downregulated across conditions in the Δ*gzf2* mutant are enriched for acyl-CoA metabolism, while upregulated genes are not associated with any specific categories of genes (Table 1). This effect is similar to that seen for wild-type *Y. lipolytica* undergoing nitrogen limitation which results in downregulation of genes with carbon response elements in the promoter region, particularly beta-oxidation genes (24).

**TABLE 1:** Enriched Gene Ontology terms for Δ*gzf2* mutant^a^

GO type	GO term	Fold enrichment	Corrected *P* value^b^
Biological pathway	Fatty acid metabolism	5.84	8.00E−08
	Phenylalanine metabolism	4.86	3.10E−04
	Glycolysis/gluconeogenesis	3.07	5.13E−04
	Fatty acid degradation	5.41	2.14E−02

Biological process	Acyl-CoA metabolic process	12.33	5.44E−07
	Oxidation-reduction process	2.04	4.96E−05
	Electron transport	3.04	2.74E−03
	Fatty acid beta-oxidation using acyl-CoA oxidase	12.33	1.36E−02

Cellular component	Peroxisome	6.66	8.47E−05
	Peroxisomal membrane	12.38	6.89E−04

Molecular function	Acyl-CoA oxidase activity	12.25	6.98E−05
	Acyl-CoA dehydrogenase activity	9.86	1.74E−04
	Flavin adenine dinucleotide binding	4.36	3.65E−03

a Analysis of top 500 up- and downregulated genes identified specific Gene Ontology (GO) terms only for genes downregulated in the Δ*gzf2* mutant (*P* < 0.05).

b *P* value was corrected for multiple comparisons using the Bonferroni method.

We suspect downregulation of genes with 5′-GYGGGG-3′ motifs in their promoters is an indirect effect mediated through carbon response element binding transcription factors. In *S. cerevisiae*, C2H2 zinc finger transcription factors, such as Mig1p, Mig2p, Mig3p, Tda9p, Rsf2p, Adr1p, Sdd4p, and YGR067Cp, interact with and regulate genes with motifs similar to GYGGGG in their promoters (57), and in *Y. lipolytica*, the homolog of Mig1p controls genes involved in beta-oxidation (58). We hypothesized that upregulation of genes with carbon response elements in their promoters is mediated by Mig1p. To test this, we identified the homolog of *mig1* as Yali0E07942g, constructed a ΔYali0E07942g strain, and sequenced RNA from cells grown under the same conditions as the GATA transcription factor mutants. We found that genes with carbon response elements are upregulated in the Δ*mig1* mutant, consistent with its role as a repressor, and opposing the effect seen in the GATA transcription factor mutants (Fig. 10). This suggests that downregulation of genes with carbon response elements in the GATA transcription factor mutants is due to increased activity of the repressor Mig1p.

## DISCUSSION

The dependence of lipid accumulation on nitrogen quality and quantity suggests an intimate regulatory link between these processes. Previous work identified amino acid metabolism as a primary controller of the response to nitrogen limitation (9) and enrichment of 5′-G(A/C)TAAGC-3′motifs in the promoters of nitrogen limitation-responsive genes as well as motifs similar to those regulated by carbon catabolite repression (24). Here we examined *Y. lipolytica* for differences in cell biology and metabolism associated with growth on a simple nitrogen source (ammonium sulfate) versus complex nitrogen source (peptone plus yeast extract) to elucidate how nitrogen quality controls lipid accumulation. Growth on the simple nitrogen source is associated with production of large lipid droplets and proliferation of peroxisomes (Fig. 1). Sequencing RNA from cells grown under the two conditions confirmed that beta-oxidation genes are upregulated on the simple nitrogen source, as would be expected if more peroxisomes were present (Fig. 6 and 8). The apparent inconsistency between high-level expression of beta-oxidation genes and accumulation of large lipid droplets is perplexing. However, flux toward triglyceride synthesis via Loa1p, Pah1p, and Dga1p must be higher than utilization of the neutral lipid pool via the triacylglycerol lipases and beta-oxidation.

We examined GATA binding transcription factors to further dissect the regulatory link between nitrogen and lipid metabolism and to test for direct and indirect regulation of lipid metabolism by regulators of nitrogen metabolism. We performed a reverse genetic screen of GATA transcription factors in *Y. lipolytica* (Fig. 2 and 3) and identified two with nitrogen source-specific phenotypes (Fig. 4) that accumulate more lipids when grown on a simple nitrogen source (Fig. 5) and characterized them by RNA sequencing (Fig. 6 to 9). We found that *gzf3* acts as a general repressor of genes with GATAA motifs in their promoter regions (Fig. 10). Interestingly, deletion of *gzf3* results in drastic overall changes to transcription level and slower growth in both conditions tested, which suggests that repression by Gzf3p is active on both simple and complex nitrogen sources. The DNA binding zinc finger domain of Gzf3p groups it with GATA repressors of nitrogen catabolism from *S. cerevisiae* (Gzf3p and Dal80p [20]) and *A. nidulans* (AreB [59]), demonstrating a general conservation of function for this repressor in *Y. lipolytica* and ascomycete fungi in general.

Deletion of *gzf2* causes a severe growth defect on a variety of simple, but not complex, nitrogen sources. Overall, the genome-wide transcription level is similar to that of the wild type in the Δ*gzf2* mutant (Fig. 8A) with the most drastic exception being genes important for nitrogen assimilation (Fig. 11). Our results show that *gzf2* is required for activation of nitrogen utilization genes but does not affect transcription as generally as the repressor (*gzf3*) and that it is not specifically required for expression of genes with GATAA sites in their promoters (Fig. 10). This suggests that activation by Gzf2p is constrained either by specific targeting to nitrogen utilization genes or is generally targeted to GATAA sites but is functional only at nitrogen utilization genes. In either case, the mechanism underlying Gzf2p’s specificity may depend on additional protein partners acting in a combinatorial manner and remains to be elucidated.

*Y. lipolytica* has two GATA transcription factors that are similar to activators of nitrogen utilization genes in *S. cerevisiae* (Gat1p [20]) and other ascomycetes (Gaf1 of *S. pombe*, AreA of *A. nidulans*, and Nit-2 of *N. crassa* [37–39]), respectively. The activator characterized here, Gzf2p, is most similar to the yeast activator Gat1p, while the second activator-like GATA transcription factor, Gzf1p, is similar to a more highly conserved group of proteins found throughout the *Ascomycota* (Fig. 3). We did not observe a particular phenotype for the Δ*gzf1* mutant (Fig. 4); however, its expression is significantly higher on the simple nitrogen source in wild-type *Y. lipolytica*, and it is regulated by both the repressor Gzf3p and the activator Gzf2p (Fig. 9), suggesting that it plays a role in nitrogen source-specific gene regulation that may be partially redundant with *gzf2*.

Modulation of nitrogen is frequently used to control lipid metabolism in oleaginous fungi (10, 13, 60), and -omic level experiments have implicated GATA binding zinc finger transcription factors as regulators of the response to nitrogen limitation in *Y. lipolytica* (9, 12). We examined *Y. lipolytica*’s complement of GATA binding transcription factors to gain a better understanding of how nitrogen metabolism is linked to lipid accumulation. Interestingly, disruption of regulators of nitrogen utilization resulted in higher lipid accumulation (Fig. 5). Metabolically, this is best explained by reduced expression of the beta-oxidation pathway in both mutants, which is regulated by carbon source-responsive transcription factors (58, 61). In both GATA mutants, we found that genes with the carbon response element GYGGGG in their promoter tend to be downregulated (Fig. 10). We attribute this to increased carbon catabolite repression mediated by *mig1*, rather than direct regulation of these genes by the GATA transcription factors. This work provides a foundation for understanding nitrogen regulation in the oleaginous yeast *Y. lipolytica* and furthers our understanding of the complex interplay between regulation of carbon and nitrogen metabolism.

## MATERIALS AND METHODS

### Yeast strains and cultivation.

All *Y. lipolytica* strains used in this study (Table 2) were maintained in YNB (1.7 g/liter yeast nitrogen base without amino acids or ammonium sulfate, 20 g/liter glucose, 5 g/liter ammonium sulfate) at 28°C and 200 rpm unless otherwise noted. Auxotrophs were supplemented with 0.1 g/liter uracil when appropriate. Frozen stocks were maintained at −80°C in 15% glycerol.

**TABLE 2:** *Y. lipolytica* strains used in this study

Strain	Genotype	Reference
FKP391	*matA leu2-270*::*leu2*^+^ *xpr2-332 axp-2 ku70*::*hph*^+^	25
FEB130	*matA leu2-270*::*leu2*^+^ *ura3 xpr2-332 axp-2 ku70*::*hph*^+^	25
FEB 56	*matA leu2-270 xpr2-332 axp-2 ku70*::*hph*^+^ *erg6*-sfGFP-*leu2*^+^	25
FEB93	*matA leu2-270 xpr2-332 axp-2 ku70*::*hph*^+^ *aim17*-sfGFP-*leu2*^+^	25
FEB64	*matA leu2-270 xpr2-332 axp-2 ku70*::*hph*^+^ *pex13*-sfGFP-*leu2*^+^	25
FEB96	*matA leu2-270 xpr2-332 axp-2 ku70*::*hph*^+^ *cpy1*-sfGFP-*leu2*^+^	25
FKP445	*matA leu2-270*::*leu2*^+^ *ura3 xpr2-332 axp-2 ku70*::*hph*^+^ *mig1*::*ura3*^+^	This work
FKP598	*matA leu2-270*::*leu2*^+^ *ura3 xpr2-332 axp-2 ku70*::*hph*^+^ *gzf3*::*ura3*^+^	This work
FKP599	*matA leu2-270*::*leu2*^+^ *ura3 xpr2-332 axp-2 ku70*::*hph*^+^ *gzf1*::*ura3*^+^	This work
FKP601	*matA leu2-270*::*leu2*^+^ *ura3 xpr2-332 axp-2 ku70*::*hph*^+^ *gzf4*::*ura3*^+^	This work
FKP602	*matA leu2-270*::*leu2*^+^ *ura3 xpr2-332 axp-2 ku70*::*hph*^+^ *gzf5*::*ura3*^+^	This work
FKP604	*matA leu2-270*::*leu2*^+^ *ura3 xpr2-332 axp-2 ku70*::*hph*^+^ *gzf6*::*ura3*^+^	This work
FKP605	*matA leu2-270*::*leu2*^+^ *ura3 xpr2-332 axp-2 ku70*::*hph*^+^ *gzf2*::*ura3*^+^	This work

### Identification and deletion of GATA transcription factors.

GATA binding transcription factors from *Y. lipolytica* were predicted using BLASTp (62) in conjunction with InterProScan (63) and Blast2GO (64). The DNA interaction domain of putative GATA binding transcription factors was identified by its Cys-X_2_-Cys-X_17_-Cys-X_2_-Cys domain and aligned to protein sequences of known GATA binding transcription factors from *S. cerevisiae*, *S. pombe*, *N. crassa*, and *A. nidulans* using MUSCLE (65). GATA transcription factors were clustered by similarity of the DNA interaction domain into families using the maximum likelihood method in MEGA6 (66). GATA transcription factor genes and *mig1* were deleted by replacement with *ura3*. Briefly, 1-kb regions flanking each gene were amplified from *Y. lipolytica* FEB130 (25) genomic DNA using Q5 DNA polymerase (New England Biolabs, Ipswich, MA) and primers (Life Technologies, Inc., Carlsbad, CA) designed with overhangs homologous to the *ura3* gene from *Y. lipolytica* strain W29 (67) (Table 3). The fragments were purified using a GeneJET purification kit (Thermo Fisher Scientific, Waltham, MA) and assembled into a deletion cassette with* ura3* using NEBuilder Hi-Fi assembly kit (New England Biolabs, Ipswich, MA). Deletion cassettes were transformed into strain FEB130 by the lithium acetate method (68), and transformants were selected on YNB agar. Replacement of genes with *ura3* was confirmed by PCR with 5′ and 3′ flanking primers followed by BamHI digestion. Deletion mutants were characterized on YNB agar at 28°C in which the 5 g/liter ammonium sulfate was replaced with an equivalent molar amount of tryptophan, urea, or 5 g/liter Bacto peptone and 5 g/liter yeast extract. Hydrogen peroxide was added to a final concentration of 4 or 6 mM in YNB agar to test for reactive oxygen species resistance.

**TABLE 3:** Primers used in this study

Primer^a^	Sequence
ura3_F	CAGTGGATCCTCTTGAGAACCGTGGAGACC
ura3_R	CAGTGGATCCCACTGTACCCAGCATCTCCG
Gzf3_5F	CCTTGGACCCCAAATTCC
Gzf3_ura3_5R	GGGTACAGTGGGATCCACTGTGAACACGTCACCAGCGT
Gzf3_ura3_3F	GTTCTCAAGAGGATCCACTGTGGGGCGTTCTATCCAAA
Gzf3_3R	CCCCCTCACCCTCAGACT
Gzf1_5F	GCTTGGCATAGGGCTGAA
Gzf1_ura3_5R	GGGTACAGTGGGATCCACTGCACCTTCACCTTCGGTGC
Gzf1_ura3_3F	GTTCTCAAGAGGATCCACTGATGAAGTCGAACGAGGCG
Gzf1_3R	GGCGCTGGATCTCAAGAA
Gzf4_5F	ACACAACCATACGGCGCT
Gzf4_ura3_5R	GGGTACAGTGGGATCCACTGCAGCTATGTGTGCACCGC
Gzf4_ura3_3F	GTTCTCAAGAGGATCCACTGGCGTCCAGTTTGACAGCC
Gzf4_3R	ATGCCAGGCCAAATTTCA
Gzf5_5F	TCAAGCCACATGGTGGTG
Gzf5_ura3_5R	GGGTACAGTGGGATCCACTGCCGTCGCTTGTTTGTGTG
Gzf5_ura3_3F	GTTCTCAAGAGGATCCACTGCTGACCTCCCACCTCCCT
Gzf5_3R	AAACCCGACTCCACACCA
Gzf6_5F	ATGGCGAGCCTCCTTTTT
Gzf6_ura3_5R	GGGTACAGTGGGATCCACTGTTGCTTGTCTGCTGCTCG
Gzf6_ura3_3F	GTTCTCAAGAGGATCCACTGGTCTGGTTGGTCGTTCCG
Gzf6_3R	AGTTGAGCACCACGCCTC
Gzf2_5F	TCGGTCTCCCGCAAATAA
Gzf2_ura3_5R	GGGTACAGTGGGATCCACTGCTCAAAATGCCCTGGGTG
Gzf2_ura3_3F	GTTCTCAAGAGGATCCACTGCAGGTTTGGTACGGGTGC
Gzf2_3R	TGCGACGGACACAAAGAA
Yali0E07942g_5F	GGGAGACTTACTGCGTGAGC
Yali0E07942g_ura3_5R	GGGTACAGTGGGATCCACTGTCCTGGGTCTTTGAGCAACT
Yali0E07942g_ura3_3F	GTTCTCAAGAGGATCCACTGGATCTGCCTGGGTGTCTGAT
Yali0E07942g_3R	GATCAACTGCCATCCCTACG

a Forward and reverse primers are indicated by the F and R letters, respectively, at the end of the primer designation.

### Growth rate determination.

*Y. lipolytica* was pregrown overnight in the medium to be assayed in, namely, either YNB or YNB-PepYe (1.7 g/liter yeast nitrogen base without amino acids and ammonium sulfate, 20 g/liter glucose, 5 g/liter Bacto peptone, 5 g/liter yeast extract). Cultures were passaged into fresh medium until they reached an optical density at 600 nm (OD_600_) of 0.001 and grown in 24-well plates in an Infinite M200 PRO microplate reader (Tecan, Mannedorf, Switzerland) at 28°C and 200 rpm. The exponential-phase growth rate was determined for three replicates of each strain in each medium by fitting log-normalized growth between OD_600_s of 0.03 and 0.1.

### Lipid quantification.

*Y. lipolytica* was pregrown overnight in either YNB or YNB-PepYe. Cultures were passaged into fresh medium to an OD_600_ of 0.05 and grown for 5 days at 28°C and 200 rpm. Total lipids were extracted using a modified form of the protocol originally developed by Bligh and Dyer (69). From each sample of *Y. lipolytica* culture, 10 ml was collected by vacuum filtration on a 0.45-μm nylon Whatman filter (GE Healthcare, Little Chalfont, United Kingdom) and washed thrice with 5 ml of yeast nitrogen base (1.7 g/liter) without amino acids and ammonium sulfate. The cells were mixed with 1.2 ml H_2_O, 3 ml methanol, and 1.5 ml chloroform in a glass vial and mixed on a rotator for 24 h. Then 1.2 ml H_2_O and 1.2 ml chloroform were added to the sample and briefly vortexed prior to separation of the organic and aqueous phases by centrifugation at 1,400 × *g* for 10 min. The organic phase was collected into a preweighed glass vial, and the extraction step was repeated three more times. The lipid-containing organic phase was dried at 40°C under a continuous stream of nitrogen gas and then weighed. Ten milliliters of culture from the same flask was separately collected and dried at 70°C to determine dry weight.

### Confocal microscopy.

For live-cell fluorescence microscopy, a panel of isogenic *Y. lipolytica* strains containing genes encoding superfolder green fluorescent protein (sfGFP)-tagged proteins specific to particular organelles were grown to an OD_600_ of 1 in either YNB or YNB-PepYe. Cells were collected, stained with calcofluor white for 5 min, and immediately visualized using a Zeiss LSM710 confocal laser-scanning microscope (Carl Zeiss MicroImaging GmbH, Munich, Germany) with a Plan-Apochromat 100× oil objective with a numerical aperture of 1.4 as described previously (70). All images were processed using ImageJ (71).

### RNA extraction, sequencing, and bioinformatic analysis.

Four replicates of each *Y. lipolytica* strain were pregrown overnight in either YNB or YNB-PepYe. Cultures were passaged into fresh medium and collected at an OD_600 _of 1. The culture was harvested by centrifugation, and RNA was extracted using Trizol (Thermo Fisher Scientific, Waltham, MA) according to the manufacturer’s instructions. Polyadenylated RNA was enriched using the NEBNext poly(A) mRNA magnetic isolation kit and prepared into an Illumina sequencing library using the NEBNext Ultra RNA library prep kit (New England Biolabs, Ipswich, MA). Single-end 50-bp reads were sequenced on an Illumina HiSeq 2500 instrument. High-throughput sequencing reads were aligned to transcripts from the *Y. lipolytica* CLIB122 genome (72) using Bowtie2 (73) and quantified using the Tuxedo Suite of tools (74). A summary of the results of the expression analysis is available in Data Set S1 in the supplemental material. Gene Ontology analysis was performed using FunRich (75). Analysis and visualization of metabolic networks utilized Cytoscape (76).
