# Supplementary figures and images for: Regulation of Nitrogen Metabolism by GATA Zinc Finger Transcription Factors in Yarrowia lipolytica
Source: mSphere. 2017 Feb 15;2(1):e00038-17. doi: 10.1128/mSphere.00038-17 (PMC5311114; doi:10.1128/mSphere.00038-17)

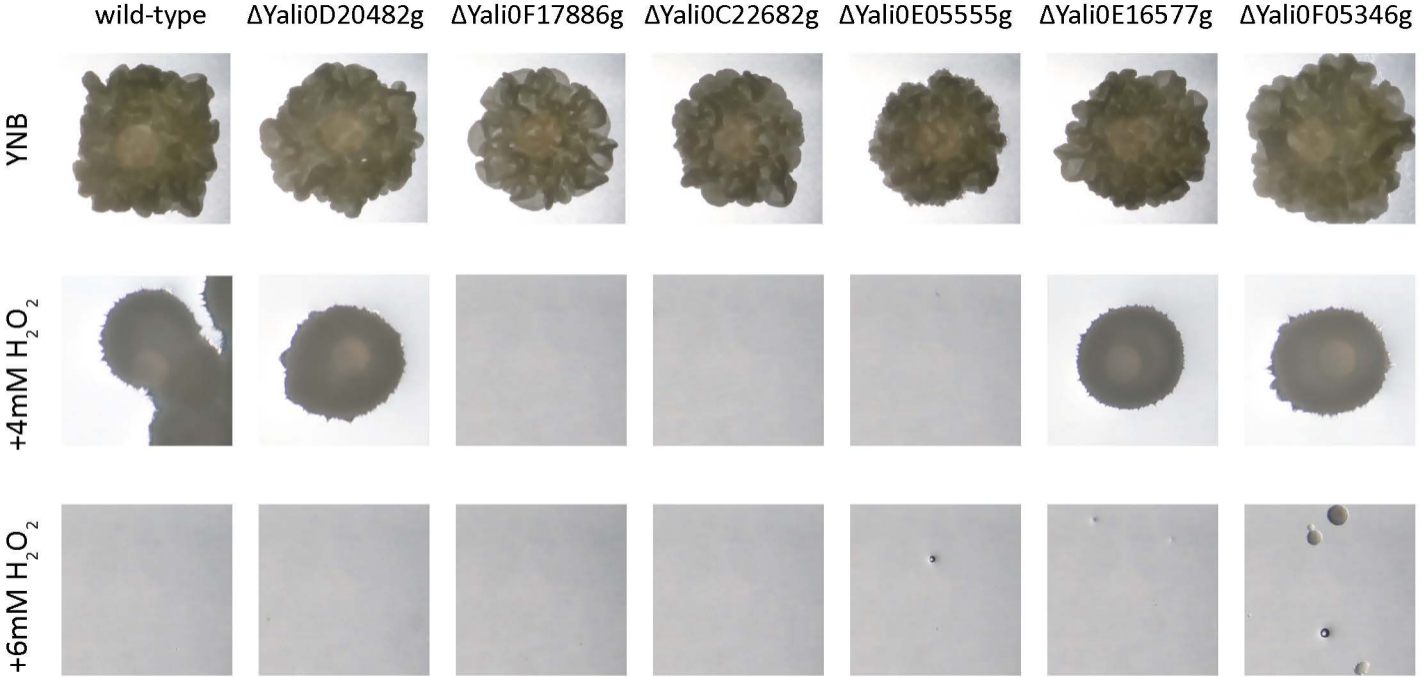

Supplement: FIG S1 [file sph001172234sf1.pdf]
